# Comparison of Genome-Wide DNA Methylation Profiles of Human Fetal Tissues Conceived by *in vitro* Fertilization and Natural Conception

**DOI:** 10.3389/fcell.2021.694769

**Published:** 2021-07-14

**Authors:** Ye Liu, Xinzhu Li, Songchang Chen, Li Wang, Yajing Tan, Xiaocui Li, Lin Tang, Junyu Zhang, Dandan Wu, Yanting Wu, Xinmei Liu, Yimin Zhu, Jianzhong Sheng, Jiexue Pan, Li Jin, Hefeng Huang

**Affiliations:** ^1^International Peace Maternity and Child Health Hospital, Shanghai Jiao Tong University School of Medicine, Shanghai, China; ^2^Key Laboratory of Reproductive Genetics (Ministry of Education), Zhejiang University, Hangzhou, China; ^3^Shanghai Key Laboratory of Embryo Original Diseases, Shanghai, China; ^4^Obstetrics and Gynecology Hospital, Institute of Reproduction and Development, Fudan University, Shanghai, China; ^5^Department of Obstetrics and Gynecology, Shanghai First Maternity and Infant Hospital, Tongji University School of Medicine, Shanghai, China; ^6^Research Units of Embryo Original Diseases, Chinese Academy of Medical Sciences, Shanghai, China; ^7^Department of Pathology and Pathphysiology, School of Medicine, Zhejiang University, Hangzhou, China

**Keywords:** IVF-ET, DNA methylation, fetal tissue, multiembryo transfer, developmental origins of health and disease

## Abstract

**Background:**

Assisted reproductive technology (ART) might induce adverse pregnancy outcomes and increase the risk of metabolic diseases in offspring’ later life with unknown reasons. Here we evaluated the global methylation level and methylation profile of fetal tissue from elective terminations of pregnancy (ETP) after natural conception and multifetal pregnancy reduction (MFPR) after *in vitro* fertilization and embryo transfer (IVF-ET).

**Results:**

Global methylation levels were comparable between the fetal tissue of ETP after natural conception group and MFPR after IVF-ET group. The methylation levels were lower in the hypermethylated regions of the MFPR group than in the ETP group, while the methylation levels were higher in the hypomethylated regions of the MFPR group. Heatmap visualization and hierarchical clustering of the candidate differentially methylated regions (DMRs) showed differences between the DMRs in the ETP and MFPR samples. We identified 196 differentially methylated regions that matched 164 genes between the ETP and MFPR groups. In the Gene Ontology (GO) and Kyoto Encyclopedia of Genes and Genomes (KEGG) pathway analyses, skeletal system morphogenesis and diabetes mellitus ranked first. Ingenuity Pathway Analysis (IPA) revealed 8 diseases and functional annotations associated with IVT-ET. In the MFPR group, the final validation showed lower methylation levels in gene bodies of bone morphogenetic protein 4 (BMP4), higher methylation levels in the 1st exon and 5′UTR of thyroid peroxidase (TPO), and higher methylation levels in TSS1500 and TSS200 of interleukin 1 beta (IL1B).

**Conclusions:**

ART does not alter global DNA methylation level, but influences DNA methylation variation in specific regions of human fetus in the early stage of life. Further studies are warranted to clarify the potential role of DNA methylation alterations in the gene expression profile.

## Introduction

The developmental origin of health and disease theory raised by David Barker points out that the period of early life is a window of developmental plasticity that is critically important for metabolic health in adulthood (Bateson et al., 2004). Developmental plasticity requires the stable modulation of gene expression, which appears to be mediated mainly by epigenetic processes, such as DNA methylation (Gluckman et al., 2008). DNA methylation in mammals is almost exclusively restricted to CpG dinucleotides. The methylation of CpG dinucleotides can repress transcription either by blocking the binding of transcription factors to the promoter or by recruiting histone-modifying protein complexes that repress transcription through the formation of a more condensed chromatin structure (Iliadou et al., 2011). Thus, learning how abnormalities in the early developmental environment interact with the physiological processes, which, via developmental plasticity, determine the patterns of adult chronic diseases, especially epigenetic changes, is essential.

Since the first assisted conception in 1978, the number of births associated with assisted reproductive technology (ART) has exceed 8 million worldwide (Calhaz-Jorge et al., 2020). Children born after ART now account for 2% of all births. Indisputable data have confirmed elevated neonatal and childhood morbidity in ART pregnancies, including preterm birth, intrauterine growth restriction (IUGR), perinatal mortality, low birth weight (LBW), small for gestational age (SGA), catch-up, congenital abnormalities, dyslipidemia, certain cancers, altered blood pressure, thyroid and cardiovascular dysfunction (Moini et al., 2012; Pandey et al., 2012; Kawwass et al., 2013; Kondapalli and Perales-Puchalt, 2013; Hu et al., 2014a; Lv et al., 2014, 2016; Xu et al., 2014; Rubens et al., 2014; Meng et al., 2015; Luke et al., 2020). The possible explanations are complex because there are many confounding factors, including ovarian stimulations, laboratory procedures manipulating gametes and embryo culture (Iliadou et al., 2011). A meta-analysis conducted by Pinborg et al. (2013) concluded that subfertility is a major risk factor for adverse perinatal outcome in ART singletons; however, factors related to hormone stimulation and/or IVF methods *per se* also may be involved. Hu et al. (2014a) indicated that serum E_2_ levels of women undergoing fresh ET at 4 and 8 weeks of gestation were significantly higher than those of women undergoing frozen ET and the women with natural conception, meanwhile, maternal high-E_2_ environment in the first trimester was correlated with increased risk of LBW and SGA. The raise of the developmental origins of adult disease has positioned low birth weight (LBW) as a significant health issue, which is tightly associated with increased risk of chronic metabolic diseases, such as metabolic syndrome, diabetes and cardiovascular disease, in later life (Lenfant, 2008; Stracquadanio and Ciotta, 2017). The exact mechanisms of these associations remain unclear; however, the epigenetic aberrations, such as abnormal genomic imprinting and methylation changes, may be involved (Ceelen et al., 2008a; Grafodatskaya et al., 2013; Pinborg et al., 2013).

In the present study, we compared the genome-wide methylation profiles of fetal tissues from cases of elective termination of pregnancy (ETP) after natural conception and multifetal pregnancy reduction (MFPR) after IVF-ET (embryo transfer) using an Illumina Infinium Human Methylation 450k BeadChip. We further validated differentially methylated CpGs/genes associated with metabolism and development in the fetal tissues. The aim of this study was to evaluate the effect of ART on the methylation profile of fetal tissues and to provide an inspiration for assessing short- and long-term health outcomes in human offspring conceived by ART.

## Materials and Methods

### Sample Collection

From January 2014 to July 2015, 12 women diagnosed with tubal-factor infertility, which became pregnant after IVF-ET undergoing MFPR [28–31 years old, 30.66 ± 0.96 (mean ± SEM)] in the International Peace Maternity and Child Health Hospital, were enrolled as the case group. Twelve age-matched women undergoing elective termination of natural pregnancy formed the control group. All enrolled women in MFPR and ETP groups had no known anatomic or genetic abnormalities. All pregnant women had a gestational age of 7–9 weeks. Mothers with severe pregnancy complications (e.g., gestational diabetes mellitus, hypertension, or abnormal thyroid function) or a family history of diabetes mellitus or other metabolic diseases were excluded. Detailed information about the study participants is summarized in Table 1. Written informed consent was obtained from all participating pregnant women. Epigenetic studies on fetal tissues were approved by the Ethics Committee of the International Peace Maternity and Child Health Hospital, Shanghai Jiao Tong University School of Medicine and the approval number is GKLW2017-81.

**TABLE 1:** Clinical baseline characteristics of the patients from whom fetal tissue DNA was derived.

Items	ETP			MFPR		
Fetal sex	Total (*n* = 12)	Female (*n* = 6)	Male (*n* = 6)	Total (*n* = 12)	Female (*n* = 6)	Male (*n* = 6)
**Age (years)**	29.45 ± 0.79	29.00 ± 1.26	29.83 ± 0.96	30.66 ± 0.96	30.00 ± 1.49	31.33 ± 1.15
**BMI (kg/m^2^)**	20.66 ± 0.56	19.93 ± 0.42	21.40 ± 0.94	20.72 ± 0.70	21.76 ± 1.02	19.69 ± 0.76
**GA (days)**	54.08 ± 0.95	55.50 ± 0.91	52.67 ± 1.47	54.45 ± 0.87	55.60 ± 1.51	53.50 ± 0.77
**E_2_ (pmol/L)**	4568.36 ± 510.83	5337.00 ± 411.14	3927.83 ± 780.56	17270.08 ± 487.13*	16675.67 ± 881.25^#^	17864.50 ± 234.15^$^
**P (pmol/L)**	114.21 ± 4.98	115.26 ± 5.23	113.33 ± 8.02	127.02 ± 0.18*	126.83 ± 0.33	127.2 ± 0

Age, maternal age; BMI, body mass index; GA, gestational age; E2, estradiol; P, progesterone. Data are presented as mean ± SEM. *P < 0.05 versus ETP Total; ^#^*P* < 0.05 versus ETP Female; ^$^*P* < 0.05 versus ETP Male.

For this study, MFPR samples were recruited from patients diagnosed with higher-order multiple pregnancies occurring as a result of IVF. The ETP and MFPR procedures were performed between 7 and 9 weeks of pregnancy, and fetal sample was acquired by using a suction unit or a 22-gauge spinal needle under negative pressure. In all cases, transabdominal ultrasound guidance was used until asystole was observed, and all procedures were performed by the same physician. The entire fetal tissues without chorionic villi were collected from ETP and MFPR and immediately stored at −80°C for subsequent analysis.

### Genome-Wide Methylation Profiling in the Fetal Tissue

The DNA from the fetal tissue (20 mg) was extracted using a DNeasy blood and tissue kit (Qiagen, Hilden, Germany) and was bisulfite-converted (500 ng of DNA per sample) using an EZ DNA methylation kit (Zymo Research, Irvine, CA, United States) according to the manufacturer’s protocol.

Genome-wide methylation profiling was performed using the Infinium Human Methylation 450k BeadChip array (Illumina, San Diego, CA, United States) according to the Illumina’s instruction. In brief, whole-genome amplification of 200 ng of input bisulfite-converted DNA was performed. The product was fragmented, purified and added to BeadChips using Illumina-supplied reagents and conditions. After the extension, the array was fluorescently labeled and scanned. The data were analyzed with GenomeStudio Methylation Module Software (Illumina). A CpG site was considered to be informative if the total signal of unmethylated and methylated sequences at the CpG loci was significantly higher (*P* < 0.05) than the signals of the negative control probes on the same array. To reduce the systematic error and batch effects, normalization control probe pairs were designed to target the same region within the housekeeping genes that did not have CpG sites in the probe. The probes were excluded if SNPs were documented in the interval covered by the Illumina probe design during hybridization. The probes were removed if they were located close (within 10 bp from the query site) to an SNP, which had a minor allele frequency of 0.05 (Emeny et al., 2018). Imputation and normalization were performed using the Subset-quantile Within Array Normalization (SWAN) method (Maksimovic et al., 2012). For each CpG site, the β value indicated the methylation level, which was calculated as β = [max (*M*, 0)]/(| U| + | M| + 100). A β value of 0–1.0 represented the methylation rate from 0 to 100%, respectively.

### Identification of Differentially Methylated Regions (DMRs)

To identify DMRs, the β values for each CpG site were calculated and normalized. The values were loaded, into limma to calculate the *P*-value of the two groups that was adjusted by Bonferroni correction. DMRs were defined after filtering for the Bonferroni correction-adjusted *P*-values of <0.05 and | Δβ| ≧20%. DMRs were also detected using “bump hunter” package into R environment (v.3.5.1) based on the genome-wide DNA methylation data. This flexible approach to data analysis effectively modeled the measurement error, removed batch effects, detected regions of interest and attached statistical uncertainty to identified regions (Jaffe et al., 2012). The principle of the analysis is based on the location on the chromosomes and their methylation regions.

Target segments were clustered based on their location (cluster nearby locations, maxGap = 1,500 bp) and the clusters were indexed by permutation test to predict statistical uncertainty of the data based on various data types, including disordered, zero-distributed, or random sampling data (bootstrap-based). Then, the segments were selected according to the cutoff value of 0.15, and permutation test was used to obtain the candidate regions by smoothing estimation of the genomic profile. In summary, bump hunting approach includes getSegments, regionFinder, linear statistical models and permutation tests to evaluate uncertainty. The output results included the start and end position on the chromosome of the DMRs, the results of clusters, indexStart, indexEnd obtained by getSegments and regionFinder, and the coefficient of the model obtained by a linear statistical model (deviation from the true value). Family-wise error rate (FWER), *P*-value and other data were provided by the permutation test.

### Functional Analysis

The Ingenuity Pathway Analysis (IPA) software (Qiagen, Hilden, Germany) was used for analyzing significant differentially methylated genes, aimed to construct a molecular interaction network, including upstream regulators, disease and biological functions and networks, using all available interaction data in the Ingenuity System Knowledge Base (IPA^1^). We predicated the expression of the differentially methylated genes. Genes with increased methylation are preset to be down-regulated and loss of methylation are preset to be up-regulated (Supplementary Material 2). A *z*-score was calculated as a predictor for the activation or inhibition state of the regulator (Kramer et al., 2014). The R package cluster Profiler process was performed for the gene functional enrichment including gene ontology (GO) and Kyoto Encyclopedia of Genes and Genomes (KEGG) (Yu et al., 2012).

### Visualization

The Circos plot was used by the Bioconductor OmicCircos package into R environment (Hu et al., 2014b). The violin graphs were plotted using ggplot2 package (Hu, 2020; Lin et al., 2021). The heatmap with the use of “heatmap” package, after data preprocessing and normalization, was plotted to identify clusters between samples.

### Bisulfite Cloning and Sequencing

For the validation of DMRs detected by the genome-wide DNA methylome analysis, genomic DNA (500 ng) was prepared using the DNeasy Kit (Qiagen, Hilden, Germany). Bisulfite conversion of the DNA was performed with the EZ DNA methylation Kit (Zymo Research, Irvine, CA, United States). Bisulfite-converted DNA (1 μL) was amplified by PCR with Taq HS DNA polymerase (Takara, Japan), and primers were designed using the MethPrimer website. DNA was cloned with the pEASY T1 Cloning Kit (Transgene, China), and the cloned DNA was digested with *Eco*RI to verify the cloning of the inserted DNA. DNA from at least ten different clones was then sequenced at BioSune Biotechnology (Shanghai) Co., Ltd. DNA methylation of individual CpG sites was analyzed using Quma software^2^.

### Statistical Analysis

Data were plotted and statistical significance was analyzed by GraphPad Prism software (version 7, La Jolla, CA, United States) or by SPSS 22.0 (SPSS, Inc., Chicago, IL, United States). The statistical significance of the differences between two groups was calculated by unpaired 2-tailed Student *t*-test. All data are shown as the mean ± SEMs. *P*-value less than 0.05 was considered significant. Statistical method for the differential methylation data has been described as previously.

### Availability of Supporting Data

The Infinium Human Methylation 450k BeadChip array data sets (raw and normalized) supporting the results and conclusions of this article are available at the NCBI Gene Expression Omnibus (GEO) repository: http://www.ncbi.nlm.nih.gov/geo/ with accession number GSE159769.

## Results

### Patient Characteristics

Table 1 shows the clinical baseline characteristics of the patients from whom fetal tissue DNA was derived. These groups were similar according to maternal age, maternal body mass index [(BMI (kg/m^2^)], and gestational age (days). The E_2_ concentration (pmol/L) was significantly higher for all MFPRs (17,270.08 ± 487.13, *n* = 12), female MFPRs (16,675.67 ± 881.25, *n* = 6), and male MFPRs (17,864.50 ± 234.15, *n* = 6) than for all ETPs (4568.36 ± 510.83, *n* = 12), female ETPs (5337.00 ± 411.14, *n* = 6), and male ETPs (3927.83 ± 780.56, *n* = 6), respectively.

### Genome-Wide Changes in DNA Methylation

**FIGURE 1:**
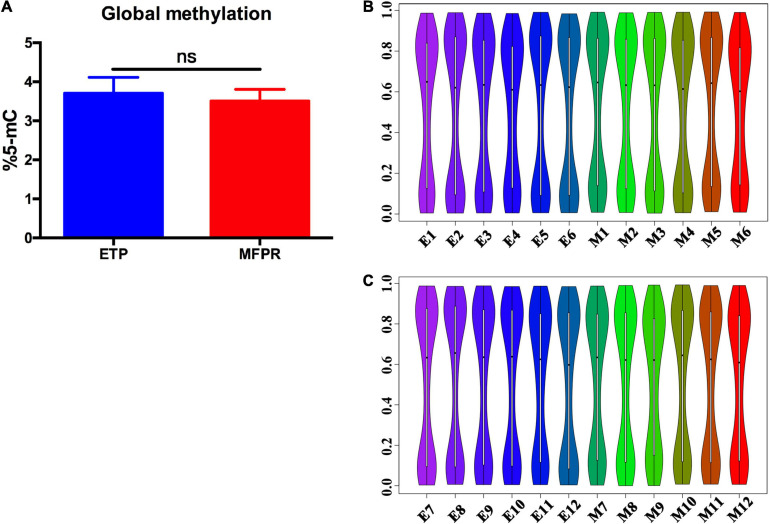
Percentage of global DNA methylation and violin plots for the overall distribution of different methylation levels in the ETP and MFPR groups. **(A)** Global DNA methylation in ETP controls (*n* = 12) and MFPR group (*n* = 12). Data are presented as mean ± SE. **(B,C)** Violin plots of the overall distribution of different methylation levels. The abscissa represents different samples, the ordinate represents the level of methylation of samples, and the width of each violin represents the density of the point at that methylation level. The boxplot shows the methylation levels in each violin, and the black dots represent the mean levels of methylation of the samples. **(B)** The overall distribution of different methylation levels in ETP male controls (left 6 violin plots labeled as E1–E6) and the MFPR male group (right 6 violin plots labeled as M1–M6). **(C)** The overall distribution of different methylation levels in ETP female (left 6 violin plots labeled as E7–E12) controls and the MFPR female group (right 6 violin plots labeled as M7–M12).

To assess DNA methylation changes associated with ART, we performed epigenome-wide association studies with the Human Methylation 450k BeadChip array using fetal tissues from the ETP and MFPR groups. This high-throughput methylation profiling technology covers 485,127 CpG sites and 99% of RefSeq genes. Collectively, the global methylation levels at all loci were not significantly different (Figure 1A). Previous research found that many factors in ART have disparate effects on offspring of different genders (Klemetti et al., 2005; Wang et al., 2013). In order to understand the reasons for gender differences, a sub-group analysis splitting the cohort by gender was performed. Violin graphs were plotted with dots representing the distributions of the CpG sites in the samples. We found that the CpG sites were more enriched in both the hypermethylated and hypomethylated regions in both sexes, with wide sections in the violin plot, but less in medium-methylated regions in both groups, with narrow sections in the violin plot (Figures 1B,C).

### Identification of Differentially Methylated Regions (DMRs)

**FIGURE 2:**
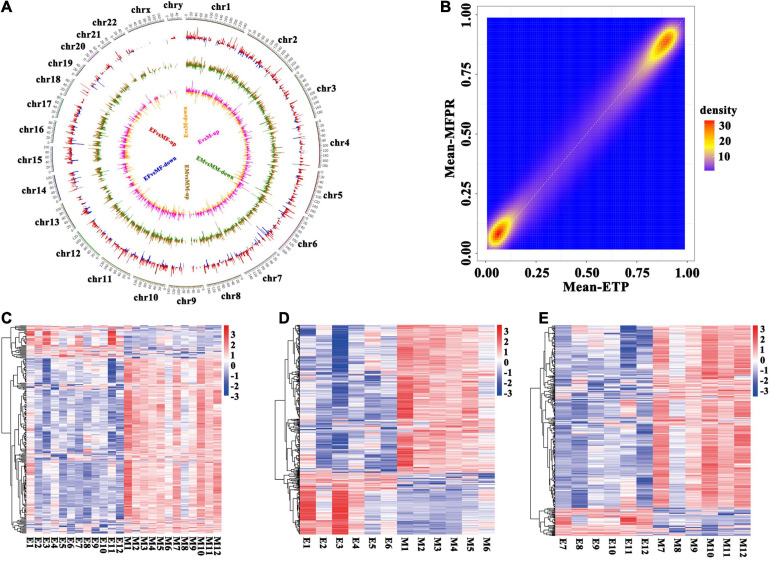
Identification of differentially methylated regions (DMRs). **(A)** Circos plots of the distribution of the whole-genome DMRs within the 23 chromosomes. From outside to inside, the first circle shows chromosome distribution with numbers representing the parts each chromosome is divided equally, the second shows the DMRs in the ETP female vs. MFPR female groups (EF vs. MF), the third shows the DMRs in the ETP male vs. MFPR male groups (EM vs. MM) and the fourth shows the DMRs in the ETP vs. MFPR groups (E vs. M). Red color indicates higher levels and blue indicates lower levels of methylation in DMRs in the MFPR group than in the ETP female group. Brown color indicates higher levels and green indicates lower levels of methylation in DMRs in the MFPR group than in the ETP male group. Pink color indicates higher levels and yellow indicates lower levels of methylation in DMRs in the MFPR group than in the ETP group. Lines represent the difference in methylation levels between the two groups, and increasing length denotes difference in methylation level. **(B)** Density plots of genome-wide paired values of fetal tissue DNA methylation. The color represents two-dimensional transformed density (blue = low density, orange = high density). **(C–E)** Heatmap cluster analysis of DMRs in the whole genome. Each row represents an individual DMR, and each column represents one group. The colors in each block from blue to white to red sequentially represent methylation ratios from –3 to 0 to 3, respectively. **(C)** Heatmap cluster analysis of DMRs in the whole genome between ETP controls and the MFPR group. **(D)** Heatmap cluster analysis of DMRs in the whole genome between ETP male controls and the MFPR group. **(E)** Heatmap cluster analysis of DMRs in the whole genome between AB female controls and the MFPR group.

To identify the patterns of differentially methylated CpGs in both groups, we used a circos plot to show the results of the whole-genome methylation analysis (Figure 2A). The circos plot revealed the distribution of the whole-genome DMRs within the 23 chromosomes in the ETP vs. MFPR groups, ETP male vs. MFPR male groups and ETP female vs. MFPR female groups and showed that mCG methylation was differentially distributed. There were few DMRs enriched on sex chromosomes. We also found that the density of the hypermethylated (>90%) regions of the MFPR group was lower than that of the ETP group, while the density of the hypomethylated (<10%) regions of the MFPR group was higher (Figure 2B). Heatmap visualization and hierarchical clustering of the candidate DMRs (| Δβ| ≧20%, *P* < 0.05) showed differences between the DMRs in the ETP and MFPR samples in the male and female subgroups (Figures 2C–E).

### Differential Methylation Near the Transcription Start Sites (TSSs) and Promoters

**FIGURE 3:**
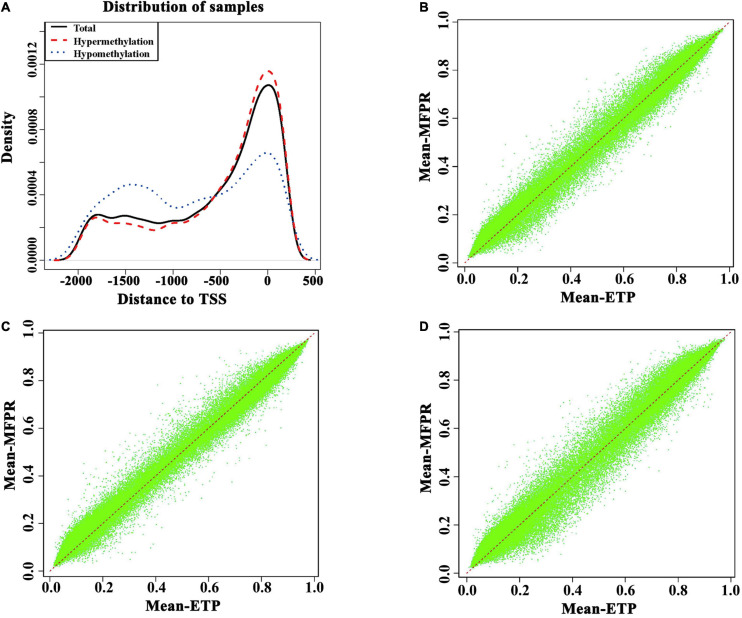
Differential methylation near transcription start sites (TSS) and promoters. **(A)** Density plots of the DMRs in TSS –2,000 to 500. Hypermethylation (red curve) represents the density curve where the mean beta is greater in the MFPR group than in the ETP controls. Hypomethylation (blue curve) represents the density curve where the mean beta is lower in the MFPR group than in the ETP controls, and total (black curve) represents the density curve at all loci. **(B)** Annotation of the promoter methylation density between the ETP vs. MFPR groups. **(C)** Annotation of the promoter methylation density between the ETP female vs. MFPR female groups. **(D)** Annotation of the promoter methylation density between the ETP male vs. MFPR male groups.

Because the DNA methylation statuses of promoters, especially the transcription start sites (TSSs), can affect gene expression through changes in chromatin structure and/or transcriptional efficiency. As shown in Figure 3A, at approximately −2,000 bp to −500 bp from the TSS, the methylation density was higher in the ETP group, and at −500 bp to +500 bp from the TSS, the methylation density was lower in the ETP group (Figure 3A). The annotations of the promoter methylation densities between the ETP vs. MFPR groups (Figure 3B), ETP female vs. MFPR female groups (Figure 3C), and ETP male vs. MFPR male groups (Figure 3D) are shown in Figures 3B–D, respectively. At the promoters, we found that the density of hypomethylated regions was higher in the MFPR group than in the ETP group, while the same trend was also evident between the ETP female and MFPR female groups. In contrast, the density of hypermethylated regions was higher in the MFPR male group than in the ETP group. We also analyzed the methylation density in the 23 chromosomes of each of the two groups, and the results indicated that the density of hypomethylated regions in each of the 23 chromosomes of the MFPR group was higher than that of the ETP group (Supplementary Figure 1).

### DMR Bumphunter Analysis

**FIGURE 4:**
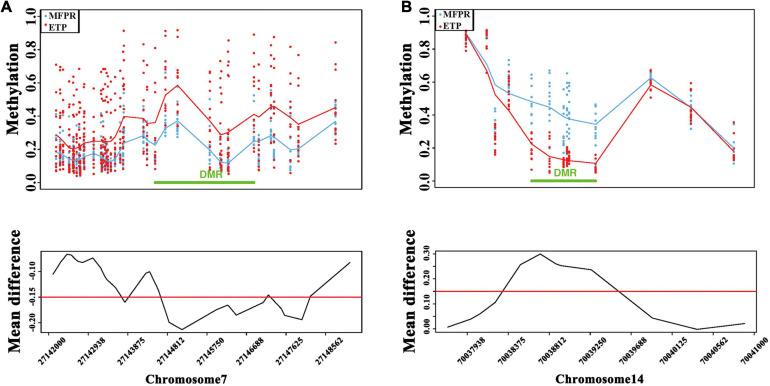
DMR bumphunter analysis. **(A)** Scatter plots of DMRs and the mean difference (cutoff value = 0.15) between ETP controls (red) vs. the MFPR group (blue) at chr7: 27142000–27148562. **(B)** Scatter plots of DMRs and the mean difference (cutoff value = 0.15) between ETP controls (red) vs. the MFPR group (blue) at chr14: 70037938–70041000.

We performed a DMR bumphunter analysis of all chromosomes and plotted chromosome methylation maps for each sample to identify DMRs. After clustering nearby locations according to maxGap (=1,500 bp), we indexed every cluster and selected segments by setting the cutoff value at 0.15. The results showed that the DMRs between two groups were mainly located on chromosomes 7 and 14 (Figures 4A,B). Although there were no significant differences in the overall levels between the two groups, we found that the methylation level was lower in the true DMRs of the MFPR group than in those of the ETP group on chromosome 7 but higher on chromosome 14 when stratified by different chromosomes.

### Gene Ontology and Kyoto Encyclopedia of Genes and Genomes Pathway Analysis of Differentially Methylated CpGs

**FIGURE 5:**
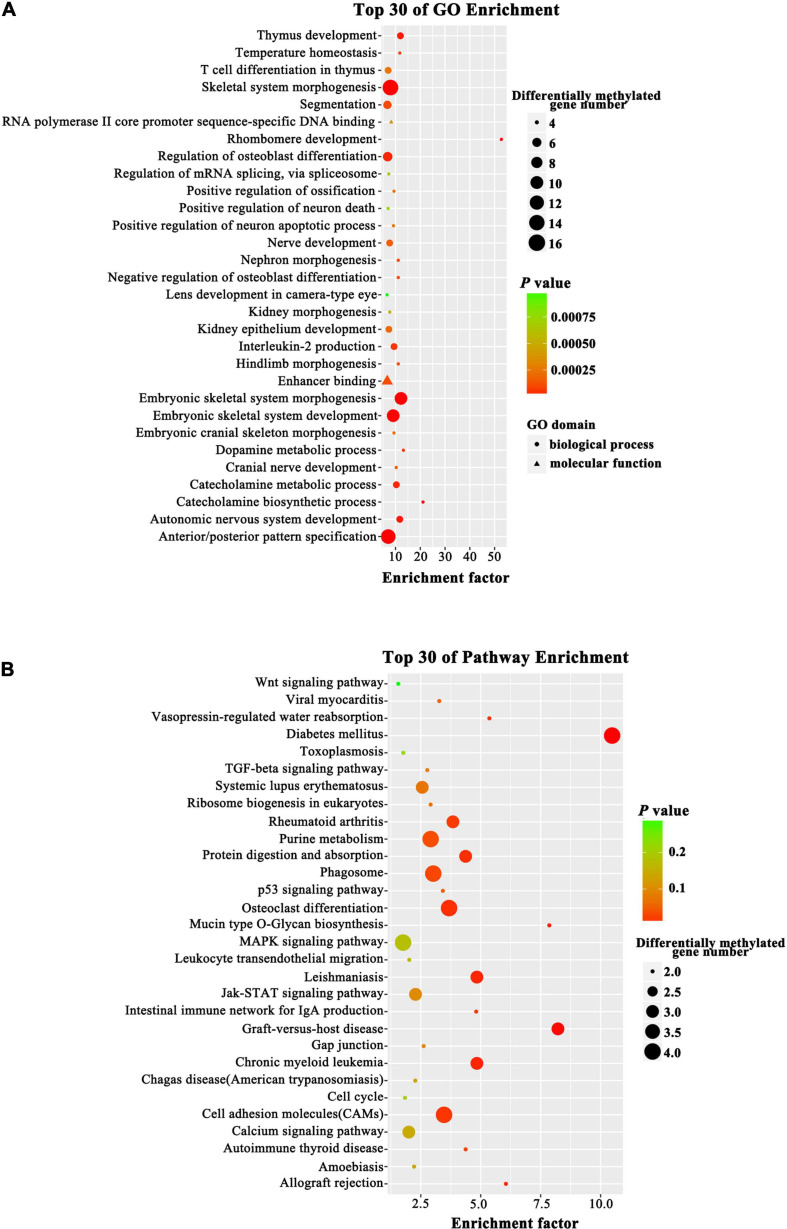
Top GO and KEGG pathway analyses of CG type DMRs. **(A)** Top GO analysis. The enrichment factor indicates the ratio of the differentially methylated gene number to the total gene number in a certain pathway. The size of each circle represents the number of genes contained in the particular class; a larger circle indicates a larger number of genes. The color indicates the *P*-value (green higher, red lower). Circles and triangles indicate biological process and molecular function, respectively. **(B)** Top KEGG analysis. The enrichment factor indicates the ratio of the differentially methylated gene number to the total gene number in a certain pathway. The size of each circle represents the number of genes contained in the particular class; a larger circle indicates a larger number of genes. The color indicates the *P*-value (green higher, red lower).

To probe changes in the methylation status of gene functions under prolificacy traits, the Gene Ontology (GO) and Kyoto Encyclopedia of Genes and Genomes (KEGG) pathway databases were analyzed to characterize the DMRs identified in our study. GO analysis revealed that DMRs were significantly enriched in the categories of embryonic skeletal system morphogenesis, embryonic skeletal system development and skeletal system morphogenesis (Figure 5A). The KEGG analysis revealed that DMRs were significantly enriched in the categories of diabetes mellitus, graft-versus-host disease, leishmaniasis, chronic myeloid leukemia and cell adhesion molecules (Figure 5B). Importantly, we found that some DMRs were involved in biological pathways that were important for development and metabolism, such as embryonic skeletal system development and diabetes mellitus, indicating that specific genes, namely, *HOXA3*, bone morphogenetic protein 4 (*BMP4*), *PTPRN2* and interleukin 1 beta (*IL1B*), which were influenced by DNA methylation.

### Ingenuity Pathway Analysis (IPA)

**FIGURE 6:**
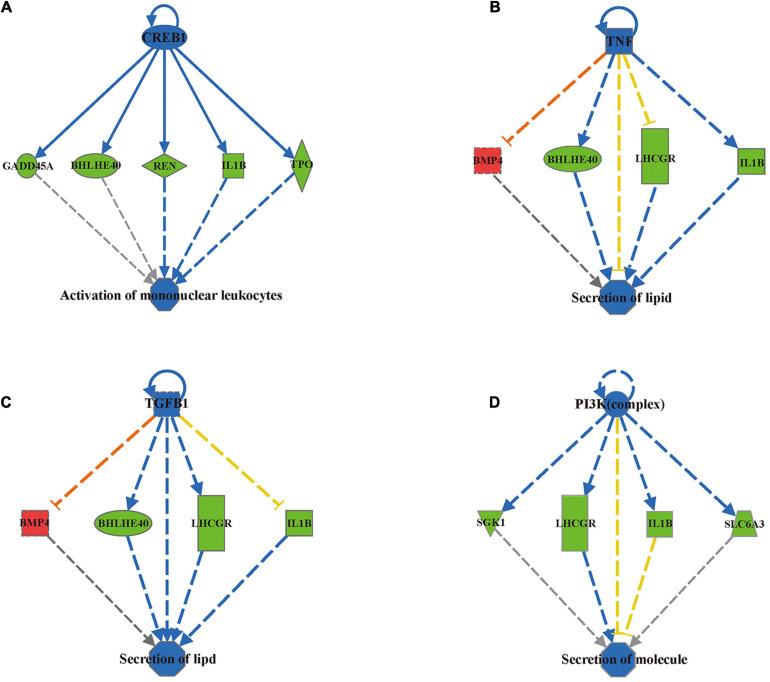
IPA predicted 4 novel regulation networks ranked by consistency score. The first layer of the network is an upstream regulatory factor, the middle layer is the differentially methylated genes uploaded from the IPA database, and the bottom layer is the related biological functions. Blue nodes indicate that the upstream regulatory factor’s function is predicted inhibition. Blue solid lines indicate a confirmed direct inhibition, while blue dashed lines indicate indirect inhibition. Red dashed lines indicate indirect activation. Yellow dashed lines indicate findings inconsistent with the state of the downstream molecule. Green nodes indicate downregulation of the expression levels of the genes and Red nodes indicate upregulation. **(A)** CREB1 regulation network. **(B)** TNF regulation network. **(C)** TGFB1 regulation network. **(D)** PI3K (complex) regulation network.

To investigate the functional relevance of the differentially methylated genes, we applied Ingenuity Pathway Analysis (IPA) and identified 196 differentially methylated regions containing 164 genes by mapping to the IPA database (Supplementary Material 2). These 196 differentially methylated regions containing 164 genes were mainly enriched in eight disease/functional annotations according to the IPA database after filtering by *z*-score < −2 or *z*-score > 2: size of body, development of body trunk, activation of lymphocytes, activation of mononuclear leukocytes, synthesis of lipid, synthesis of steroid, secretion of lipid and secretion of molecule (Table 2). To determine how differential DNA methylation contributes to the pathogenesis of adverse outcomes after IVF-ET, novel regulation networks were generated from IPA based on the regulation network integrity analysis. The top 4 networks, which were ranked by consistency score, are shown in Figure 6. From the novel regulation network constructed by IPA, we speculate that *CREB1*, *TNF*, *TGFB1*, and *PI3K* are upstream regulators that control the expression of downstream differentially methylated genes, including *BMP4*, *IL1B*, and thyroid peroxidase (*TPO*). This process may alter the related biological functional pathways and impact the activation of mononuclear leukocytes and the secretion of lipids and molecules, causing disease in later life.

**TABLE 2:** Eight disease/functional annotations according to the IPA database.

Diseases or functions annotation	*p*-value	Predicted activation state	Activation *z*-score	Molecules
**Size of body**	1.17E-02	Decreased	−2.545	APBA2,**BMP4**,CD9,CPLX1,CSGALNACT1,DBH,HDAC4,HOXD4,LHCGR,LTBP3, PARK2,POSTN,REN,SLC6A3,STARD13,WWOX
**Development of body trunk**	1.98E-03	Decreased	−2.531	ARID3B,ATF7,BCL11B,**BMP4**,CD9,FOXP1,HDAC4,HOXA3,IL1B,JARID2,LHCGR, MAD1L1,NCOR2,NRG4,PTPRN2,REN,RLTPR,RPTOR,SCHIP1,STARD13,VCAN
**Activation of lymphocytes**	1.24E-02	Decreased	−2.400	BCL11B,BHLHE40,GADD45A,HDAC4,**IL1B**,IL21R,NCOR2,NFKBIZ,REN,**TPO**
**Activation of mononuclear leukocytes**	6.48E-03	Decreased	−2.391	BCL11B,BHLHE40,FOXP1,GADD45A,HDAC4,**IL1B**,IL21R,NCOR2,NFKBIZ,REN,**TPO**
**Synthesis of lipid**	4.57E-03	Decreased	−2.335	BMP4,C1QTNF3,CCHCR1,CD9,CERS6,CNTFR,CUX1,DEGS2,**IL1B**,LHCGR,LPIN1,REN,RPTOR,TBXAS1,WWOX
**Synthesis of steroid**	2.14E-03	Decreased	−2.115	BMP4,C1QTNF3,CCHCR1,**IL1B**,LHCGR,REN,TBXAS1,WWOX
**Secretion of lipid**	4.43E-03	Decreased	−2.052	BCL11B,BHLHE40,**BMP4**,**IL1B**,LHCGR,PTPRN2
**Secretion of molecule**	2.25E-03	Decreased	−2.024	BCL11B,BHLHE40,**BMP4**,C1QTNF3,CASP4,CPLX1,DBH,**IL1B**,LHCGR,PARK2, PTPRN2,SGK1,SLC6A3

*Bold indicates validated genes.*

### Validation of the Candidate Differentially Methylated Genes

**FIGURE 7:**
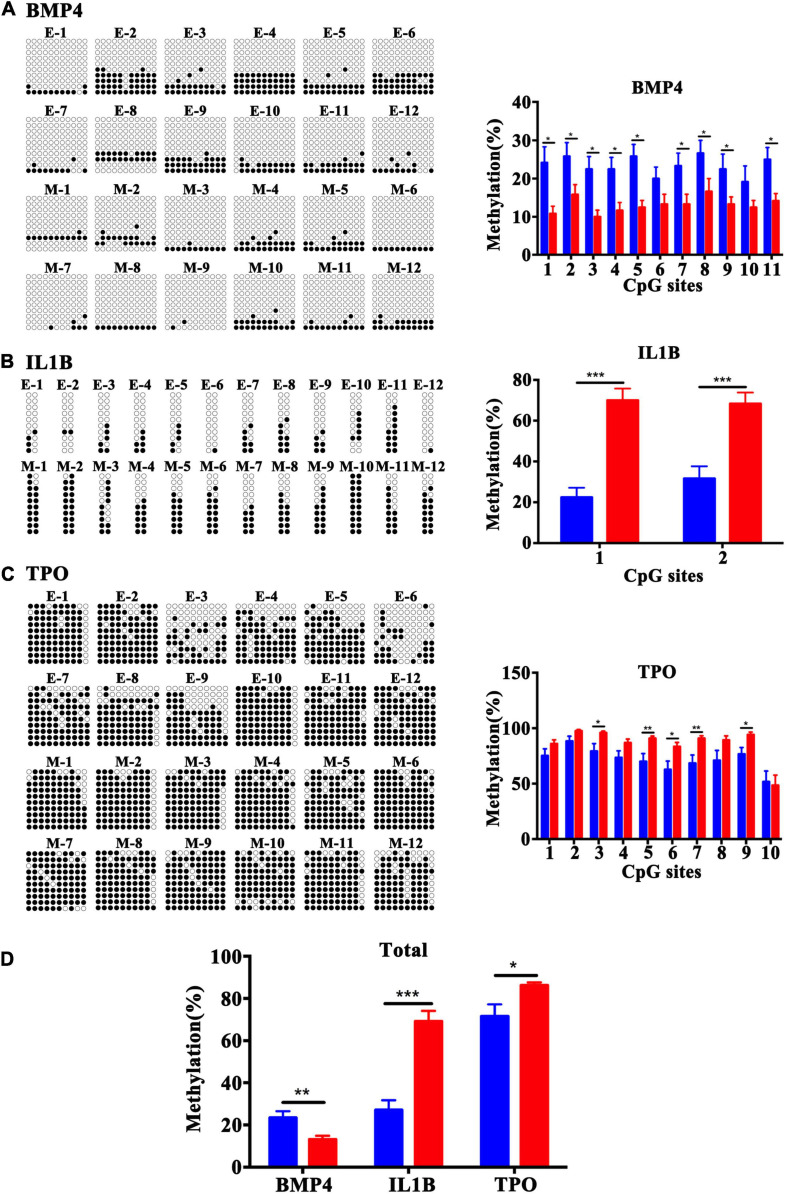
Validation of the methylation levels by bisulfite sequencing in BMP4, IL1B, and TPO DMRs at base-pair resolution. Open circles and filled circles represent unmethylated and methylated CpG sites, respectively. **(A)** Bisulfite sequencing data for 11 CpG sites at the gene bodies of BMP4. **(B)** Bisulfite sequencing data for 2 CpG sites in the TSS1500 to TSS200 regions of IL1B. **(C)** Bisulfite sequencing data for 10 CpG sites at the 1st exon and 5′UTR of TPO. **(D)** Total methylation rate of BMP4, IL1B and TPO. The blue square represents the ETP group and red represents the MFPR group. Data are presented as mean ± SEM. *p < 0.05; **p < 0.01; ***p < 0.001.

To validate the array results, three candidate genes, *BMP4, TPO*, and *IL1B*, were selected. Bisulfite cloning and sequencing of these three genes was performed to validate the DMRs at a base-pair resolution in a separate set of samples [fetal tissues were from women who had similar maternal age, maternal BMI (kg/m^2^) and gestational age (days)]. The DMRs in the *BMP4* gene were confirmed to be hypomethylated at the CpG sites 1, 2, 3, 4, 5, 7, 8, 9, and 11 (Figure 7A), and the DMRs in the TSS1500 and TSS200 of the *IL1B* gene (Figure 7B) and in exon 1 and 5′UTR of the *TPO* gene (Figure 7C) were hypermethylated at the CpG sites 1 and 2 and CpG sites 3, 5, 6, 7, and 9, respectively, in the MFPR group. Validation by bisulfite cloning and sequencing showed that the methylation changes in *BMP4*, *TPO*, and *IL1B* DMRs were consistent with the results of the Infinium Human Methylation 450 BeadChip array of each gene (Figure 7D). These findings confirmed the array results.

## Discussion

Whether ART impacts the epigenome and the long-term health of offspring is still controversial. One possible reason is lack of understanding of the epigenetic contribution of ART to the offspring rather than confounding factors during pregnancy. To date, studies that have investigated the effects of ART on DNA methylation in humans have mainly focused on a limited number of loci, usually known as key regions associated with imprinting disorders (Lou et al., 2014; Sakian et al., 2015; Vincent et al., 2016), and only four studies have compared the genome-wide methylation profiles in umbilical cord blood samples and placenta after natural conception and ART. The study conducted by Katari et al. (2009) described CpG methylation in the promoter regions of 1,536 CpG sites, primarily associated with imprinting, from the cord blood and placentas of 10 children conceived *in vitro* and 13 children conceived *in vivo* using a bead-array platform (GoldenGate Array, Illumina, Inc., United States). The results suggested that *in vitro* conception is associated with lower methylation at CpG sites in placenta and higher methylation at CpG sites in cord blood (Katari et al., 2009). Melamed et al. (2015) used a genome-wide approach with the Illumina^®^ Infinium Human Methylation27 array and interrogated 27,578 CpG sites in cord blood samples from ART (*n* = 10) and control pregnancies (*n* = 8). They observed that 733 of the CpG sites were significantly differentially methylated between the two groups, with an overall relative hypomethylation in the ART group (Melamed et al., 2015). Recently, a study from the United States investigated the neonatal blood spots of 137 newborns conceived spontaneously, through intrauterine insemination (IUI), or through ICSI using fresh or cryopreserved (frozen) embryo transfer using the Illumina Infinium Human Methylation 450k BeadChip and revealed that both infertility and ICSI altered DNA methylation at specific genomic loci, an effect that was mitigated to some extent by FET (Estill et al., 2016). Another study conducted by El Hajj et al. (2017) obtained umbilical cord blood samples from 48 ICSI and 46 control pregnancies for genome-wide analyses with Illumina’s 450K methylation arrays. The DMRs identified in those studies did not overlap/align with those identified in our study. The possible explanations of this is the different gestational age. In our study, we only recruited the fetus samples with gestational age of 7–9 weeks. It is well known that the embryonic period is critical for methylation remodeling influenced by a number factors, including intrauterine environment, maternal diet and lifestyle habits. Therefore, after the long-term effect of perinatal period, numerous confounding factors contribute to the analysis of perinatal samples, not only the ART itself. So samples from early stage of gestational age are of great significance to evaluate the effect of early stage event on offspring. And the samples used in these studies were limited to perinatal umbilical cord blood and placenta; thus, the observed methylation changes might not truly reflect the epigenomic changes in the offspring.

The objective of our study was to obtain a full picture of the methylation pattern and novel insights into the epigenetic mechanisms that underlie abnormal outcomes after IVF-ET in samples minimizing the effect of the perinatal environment on DNA methylation. We compared the genome-wide methylation of fetal tissues from cases of ETP and MFPR for the first time using the Infinium Human Methylation 450k BeadChip array and found numerous differences. As we showed (Figure 1A), ART did not influence global DNA methylation status, consistent with previous finding conducted by Novakovic et al. (2019). This study performed a longitudinal epigenome-wide association study (EWAS) in the blood collected from the neonates and adults (age 22–35 years) and reported no evidence of a significant effect of ART on global offspring’s methylation change (Novakovic et al., 2019). However, we identified 196 differentially methylated regions that matched 164 genes altered in the fetal tissues of the MFPR group compared to the ETP group. The hierarchical clustering presentations (Figures 2C–E) indicate that consistency of the data is well. GO and KEGG pathway analyses of 164 differentially methylated genes revealed that skeletal system morphogenesis and diabetes mellitus may be involved in the abnormal outcomes in offspring after IVF-ET. Additionally, IPA revealed the top 8 disease and functional annotations, such as size of body, synthesis of lipid and synthesis of steroid, associated with IVF-ET. The results of the bisulfite sequencing showed that the methylation levels of *BMP4, TPO*, and *IL1B* were in accordance with the Infinium Human Methylation 450 BeadChip array analysis.

The epigenomes of sperm, oocytes and embryos are reprogrammed during gametogenesis and embryogenesis to establish full developmental potential. ART procedures primarily consist of artificial hormones, superovulation, *in vitro* manipulation, culture of germ cells and preimplantation embryos during a critical period of the genome-wide epigenetic demethylation and reprogramming (El Hajj and Haaf, 2013; Zhu et al., 2018); each of these steps may play a role in impacting the DNA methylation of offspring after ART. As is shown in Table 1, we analyzed the maternal serum levels of E_2_ before elective terminations of pregnancy and reduction in multiple pregnancies, and found that the level of E_2_ in the MFPR group was higher than that in the ETP group. These results suggest that ART procedures could induce supraphysiological E_2_ environment during embryo development. Gametes and embryos at this stage are highly susceptible to external factors, and their demethylation and reprogramming are easily disturbed. In the present study, we used fetal tissues in the first trimester to minimize the effect of intrauterine and perinatal environment on epigenetic modifications. Our result indicated that ART procedure did not change the global methylation level of the offspring, however, the DNA methylation patterns of the offspring from IVF-ET and natural conceptions were different. However, this study has certain limitations. We used the entire fetal tissue obtained from both groups to extract DNA for subsequent methylation analysis because of the approach we used to collect the samples, which may lead to sample bias. We presume that these DNA samples could reflect the overall methylation level of the embryo. On the other hand, samples obtained after reduction in multiple pregnancies in the MFPR group may be biased compared with samples in single pregnancy in the control group. This fact is determined by the difficulty in obtaining early embryonic tissue from natural multiple pregnancies which are very rare. It is more convincible if the sample size is larger (Tsai and Bell, 2015). Studies based on larger sample size will provide additional support to our results due to considerable individual differences in clinical samples. However, from our point of view, our findings provide new insight into the epigenetic mechanisms associated with health outcomes of IVF-born children. Future studies of IVF-associated methylation changes in the human fetal tissue using improved methods to compensate for sample bias may help to determine these changes are caused by IVF itself.

We observed a large number and genome-wide distribution of DMRs throughout the whole genome in heatmaps, indicating that ART procedures may influence the DNA methylation patterns of offspring. Evidence from recent studies has suggested that the genomic location of DNA methylation is a major contributor to the type of functions performed by this epigenetic modification. For example, in gene promoters, methylated CpGs can prevent the binding of some transcription factors or change the structure of the chromosome to downregulate gene expression. Intergenic sequences contain enhancers and insulators that are associated with the regulation of gene expression during differentiation and organogenesis, which can be easily affected by epigenetic modulation (Jones, 2012; Schubeler, 2015). DNA methylation in introns can modulate alternative exon splicing (Maunakea et al., 2010; Shukla et al., 2011; Wang et al., 2015). In CpG island shores, differential DNA methylation is tissue-specific and may regulate transcription from alternative start sites (Irizarry et al., 2009). Because of the influence of promoter methylation status on gene expression, we only patterned the distribution of DMRs located at −2,000 to 500 bp from TSS in promoters. We found that at approximately −2000 to −500 bp from the TSS, the methylation density was lower in the MFPR group than in ETP control, while at −500 to +500 bp from the TSS, the methylation density was higher in the MFPR group. To the best of our knowledge, methyl-CpG binding domain (MBD) proteins are key molecules in the interpretation of DNA methylation signals, leading to gene silencing through the recruitment of chromatin remodeling complexes. Among them, MBD2-binding sites near TSSs have a prominent role in gene silencing. Interestingly, a distance effect was observed for binding sites located both upstream and downstream from the TSS. The results of Chatagnon’s study demonstrated that the association between MBD2 binding and transcriptional repression is weakened if the distance between the binding site and TSS is increased. These authors found that the relative MBD2 signal probe-by-probe analysis started to increase −700 bp upstream from a TSS, peaking between 0 and −200 bp downstream from the TSS (Chatagnon et al., 2011). In our study, the methylation density was higher in the MFPR group than in the ETP control at −500 bp upstream to +500 bp downstream, suggesting that MBD2 binds to sites near TSSs more frequently in the MFPR group than in the ETP group, causing gene silencing effects. Hence, this binding may be an important epigenetic mechanism underlying short-term and long-term effect for ART offspring.

Increasing evidence shows that ART is associated with adverse perinatal outcomes, birth defects, cancers and long-term chronic aging related diseases (Rimm et al., 2004; Allen et al., 2006; Scherrer et al., 2012; Hargreave et al., 2013; Chen et al., 2014), and epigenetic effects are likely to represent one of the most important underlying mechanisms. Ceelen et al. (2009) conducted a study including 392 children (*n* = 193 IVF, *n* = 199 control) and collected growth data from birth to 4 years of age. They found that children born after IVF-ET had significantly lower weight, height and BMI at 3 months than controls. Likewise, IVF offspring demonstrated catch-up growth during late infancy (3 months to 1 year) (Ceelen et al., 2009). In our study, GO and KEGG pathway analyses of the differentially methylated CpGs in the fetal tissues of MFPR groups showed that the hypermethylation of CpGs in promoters occurred in the pathway of embryonic skeletal system morphogenesis and development. IPA analysis showed that the biological processes of size of body and development of body trunk were predicted to be suppressed in the MFPR group. These results indicated that the altered methylation status in the IVF fetus may be an important molecular mechanism leading to lower weight, height and BMI. To date, the results of several studies have indicated that ART may also impair glucose and lipid metabolism in offspring. Ceelen et al. (2008b) examined fasting glucose levels in 225 IVF-conceived children and 225 age- and sex-matched spontaneously conceived control children. They reported higher fasting glucose levels in pubertal IVF children than in controls (Ceelen et al., 2008b). We also found that differentially methylated sites in IVF fetal tissues were enriched in the glucose metabolism pathway through GO and KEGG analysis, which may be one of the potential mechanisms leading to impaired glucose tolerance. Sakka et al. (2010) reported that children born after IVF-ET had significantly higher triglyceride levels but normal total cholesterol, high-density lipoprotein and low-density lipoprotein levels. Through IPA analysis, we found the differential methylation of genes enriched in biological process categories such as synthesis of lipid, synthesis of steroid, secretion of lipid and secretion of molecule, which provided a possible explanation of the abnormal lipid metabolism of IVF progeny. A meta-analysis conducted in 2013 showed that increased risks for leukemias (RR = 1.65; 95% CI, 1.35–2.01), neuroblastoma (RR = 4.04; 95% CI, 1.24–13.18), and retinoblastomas (RR = 1.62; 95% CI, 1.12–2.35) were associated with fertility treatment (Hargreave et al., 2013; Luke et al., 2020). Consistent with our predictions, we found that differentially methylated sites were enriched in the pathways of chronic myeloid leukemia, activation of lymphocytes, and activation of mononuclear leukocytes by GO/KEGG and IPA analyses, which may cause immunological abnormalities, providing a possible explanation for increased incidence of cancer in IVF progeny. Among the relevant genes in predicted affected pathways by IPA analysis, *BMP4* was noted for its vital roles in organismal development, embryonic development, cell-to-cell signaling and interaction, lipid metabolism and molecular transport. Being a crucial factor for embryonic skeletal system morphogenesis and development pathways, *BMP4* was found to be hypomethylated in gene bodies, and the average methylation level was decreased to 10% in our study. In the predicted novel regulatory networks, *CREB1*, *TNF*, *TGFB1*, and *PI3K* are predicted by IPA to be upstream negative regulators that modulate the expression of downstream differentially methylated genes such as *BMP4*, *IL1B*, and *TPO*. This process may alter related biological functional pathways and impact the activation of mononuclear leukocytes and the secretion of lipids and molecules, causing disease among IVF children in later life. This process also produces predictions of the risks of adult diseases in those born after IVF-ET. Many studies reported that DNA methylation status had a strong inverse correlation with gene expression, but that is not always the case in reality (Jones, 2012; Yin et al., 2017). The expression level and following GO and KEGG pathway analysis are predicted. Future combined analysis of transcriptome and methylation analysis will provide more insights.

## Conclusion

Overall, we detected epigenetic signatures in fetal tissues after IVF-ET with the use of a genome-wide methylation array, affecting 0.11% of CpG sites. Through bioinformatics analysis, we predicted important genes and pathways that are susceptible during reprogramming when using ART. These findings provide new insights into the underlying epigenetic mechanisms for health outcomes of IVF offspring. In the future, predictive epigenetic markers of therapeutic interventions should be developed and ART children should be systematically tracked to optimize the long-term health of IVF children.

## Data Availability Statement

The datasets generated for this study can be found in online repositories. The names of the repository/repositories and accession number(s) can be found below: https://www.ncbi.nlm.nih.gov/, GSE159769.

## Ethics Statement

The studies involving human participants were reviewed and approved by the Ethics Committee of the International Peace Maternity and Child Health Hospital, Shanghai Jiao Tong University School of Medicine and the approval number is GKLW2017-81. The patients/participants provided their written informed consent to participate in this study.

## Author Contributions

YL, XZL, and JP performed the experiments and wrote the manuscript. HH and LJ developed the experimental designs. JP, YW, XML, YZ, and JS participated in the study conception and design. SC, LJ, LW, YT, and XCL collected the samples used in the study. LT performed the assays of indicators in serum. JZ and DW analyzed the data. HH was the guarantor of this work and, as such, had full access to all data in the study and takes responsibility for the integrity and accuracy of data analysis. All authors read and approved the final manuscript.

## Conflict of Interest

The authors declare that the research was conducted in the absence of any commercial or financial relationships that could be construed as a potential conflict of interest.

## Footnotes

1 http://www.ingenuity.com

2 http://quma.cdb.riken.jp/

## Supplementary Material

Supplementary Figure 1 Annotation of the promoter DMR methylation density in 23 chromosomes between the ETP vs. MFPR groups.

Supplementary Material 2 List of 196 differentially methylated regions that were matched to 164 genes.

Supplementary Material 3 Normalized data of genome-wide DNA methylation profiles.
