# Supplementary figures and images for: Comparison of Genome-Wide DNA Methylation Profiles of Human Fetal Tissues Conceived by in vitro Fertilization and Natural Conception
Source: Front Cell Dev Biol. 2021 Jul 14;9:694769. doi: 10.3389/fcell.2021.694769 (PMC8318003; doi:10.3389/fcell.2021.694769)

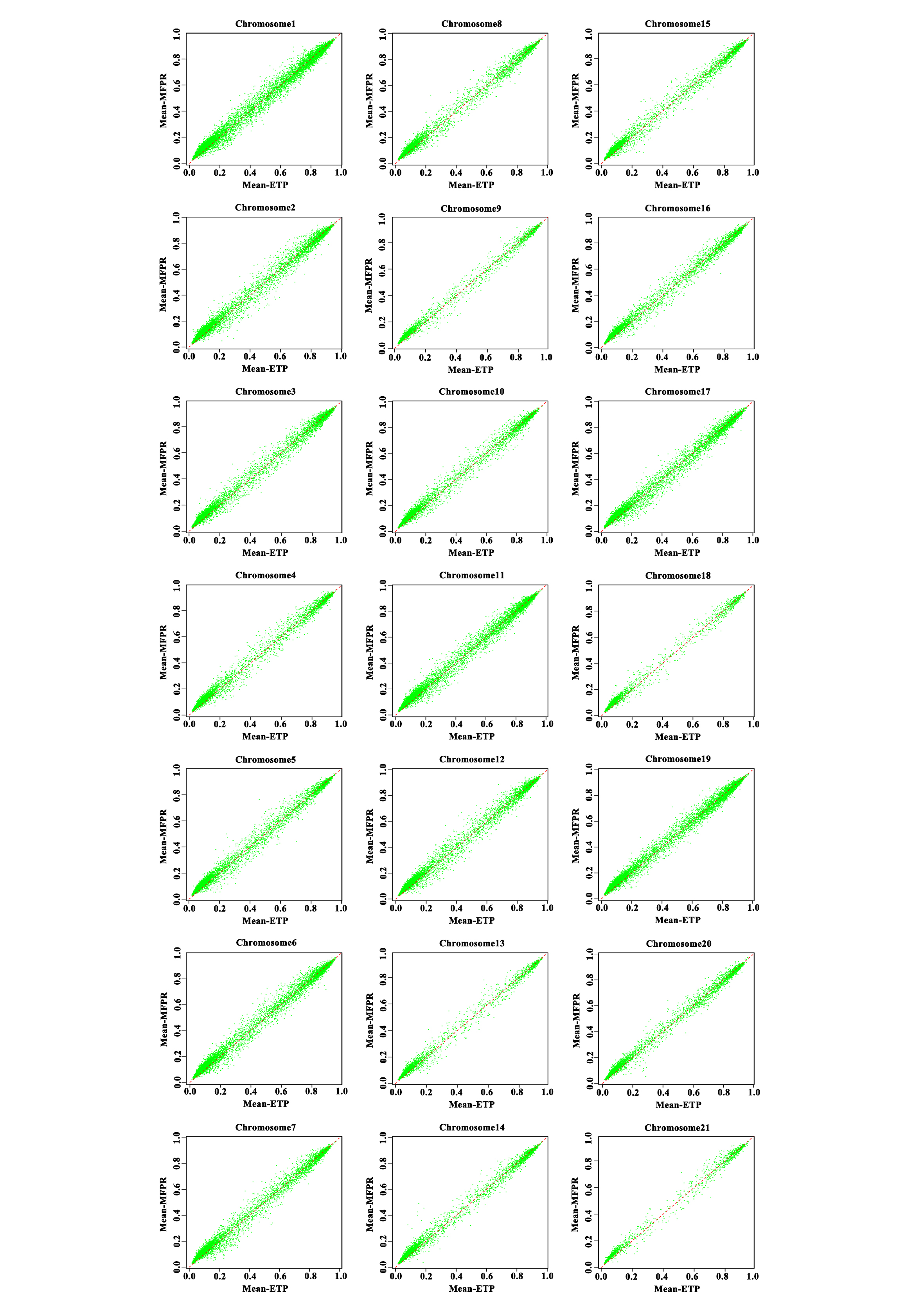

Supplement: Supplementary Figure 1 — Annotation of the promoter DMR methylation density in 23 chromosomes between the ETP vs. MFPR groups. [file Image_1.JPEG]
